# The challenges in monitoring and preventing patient safety incidents for people with intellectual disabilities in NHS acute hospitals: evidence from a mixed-methods study

**DOI:** 10.1186/1472-6963-14-432

**Published:** 2014-09-24

**Authors:** Irene Tuffrey-Wijne, Lucy Goulding, Vanessa Gordon, Elisabeth Abraham, Nikoletta Giatras, Christine Edwards, Steve Gillard, Sheila Hollins

**Affiliations:** Faculty of Health, Social Care and Education, St. George’s University of London and Kingston University, 2nd floor Grosvenor Wing, Cranmer Terrace, London, SW17 0RE UK; King’s Improvement Science, King’s College London, De Crespigny Park, London, SE5 8AF UK; NHS England Patent Safety Maple Street, London, W1T 5HD UK; Florence Nightingale Faculty of Nursing and Midwifery, King’s College London, James Clerk Maxwell Building, 57 Waterloo Road, London, SE1 8WA UK; Cass Business School, City University London, London, EC1Y 8TZ UK; Institute of Leadership and Management in Health, Kingston University Business School, Kingston, UK; Institute of Population Health, St George’s University of London, Cranmer Terrace, London, SW17 0RE UK

**Keywords:** Intellectual disability, Learning disability, Patient safety, Hospital, Health Services Research, Safety culture, Quality improvement

## Abstract

**Background:**

There has been evidence in recent years that people with intellectual disabilities in acute hospitals are at risk of preventable deterioration due to failures of the healthcare services to implement the reasonable adjustments they need. The aim of this paper is to explore the challenges in monitoring and preventing patient safety incidents involving people with intellectual disabilities, to describe patient safety issues faced by patients with intellectual disabilities in NHS acute hospitals, and investigate underlying contributory factors.

**Methods:**

This was a 21-month mixed-method study involving interviews, questionnaires, observation and monitoring of incident reports to assess the implementation of recommendations designed to improve care provided for patients with intellectual disabilities and explore the factors that compromise or promote patient safety. Six acute NHS Trusts in England took part. Data collection included: questionnaires to clinical hospital staff (n = 990); questionnaires to carers (n = 88); interviews with: hospital staff including senior managers, nurses and doctors (n = 68) and carers (n = 37); observation of in-patients with intellectual disabilities (n = 8); monitoring of incident reports (n = 272) and complaints involving people with intellectual disabilities.

**Results:**

Staff did not always readily identify patient safety issues or report them. Incident reports focused mostly around events causing immediate or potential physical harm, such as falls. Hospitals lacked effective systems for identifying patients with intellectual disabilities within their service, making monitoring safety incidents for this group difficult.

The safety issues described by the participants were mostly related to delays and omissions of care, in particular: inadequate provision of basic nursing care, misdiagnosis, delayed investigations and treatment, and non-treatment decisions and Do Not Attempt Cardiopulmonary Resuscitation (DNACPR) orders.

**Conclusions:**

The events leading to avoidable harm for patients with intellectual disabilities are not always recognised as safety incidents, and may be difficult to attribute as causal to the harm suffered. Acts of omission (failure to give care) are more difficult to recognise, capture and monitor than acts of commission (giving the wrong care). In order to improve patient safety for this group, the reasonable adjustments needed by individual patients should be identified, documented and monitored.

## Background

### Patient safety

Patient safety for people with intellectual disabilities accessing healthcare services has been an area of increasing concern in recent years. Patient safety is the prevention of errors and adverse effects to patients associated with health care [1]. A patient safety incident is any healthcare related event that was unintended, unexpected and undesired and which could have or did cause harm to patients [2]. Examples include acquisition of a hospital-acquired infection, receiving the wrong medication (or the wrong route of administration of medication), or falls whilst under the hospital’s care. In analysing patient safety incidents, one aspect to be considered is the impact on the patient, which can range from no known harm to severe harm or death; another is the likelihood of a similar incident happening again [3]. Those incidents that result in harm are termed ‘adverse events’. A sub-set of patient safety incidents are described as ‘never events’ , which are the most serious, largely preventable patient safety incidents that should not occur if the available preventative measures had been implemented by healthcare providers [4].

It is estimated that one in ten patients experience adverse events in hospital [1, 5], and that around 50% of these are preventable [6]. Whilst human error can never be completely eradicated, there are often a number of different ‘contributory factors’ , spanning cross-organisational, organisational and individual levels, which underpin adverse events. There is a consensus among patient safety advisors that the best way of improving reporting and reducing harm is to target the underlying systems failures rather than take action against individual members of staff [7].

### Patient safety of people with intellectual disabilities

Intellectual disabilities (also known as learning disabilities in the UK) are defined as ‘a significantly reduced ability to understand new or complex information, to learn new skills (impaired intelligence), with a reduced ability to cope independently (impaired social functioning) which started before adulthood, with a lasting effect on development’ [8] (p14). Approximately 2% of the population have intellectual disabilities [9].

The health inequalities faced by people with intellectual disabilities are well evidenced; they have poorer health than the general population and need to use health services more frequently [10–15].

There is evidence that certain groups may be more at risk of patient safety incidents due to their vulnerable status [16]. In 2004 the National Patient Safety Agency (NPSA) investigated the safety issues experienced by people with intellectual disabilities within the NHS. They suggested that the following put this group of patients at higher risk of potential or actual harm: high levels of comorbidities; late diagnosis and risk of ‘diagnostic overshadowing’ (where symptoms are erroneously attributed to the intellectual disability, rather than to a physical illness); communication breakdowns; failures in the provision of personal care; and issues around mental capacity and decision making [17]. The NPSA suggested that patients with intellectual disabilities are vulnerable to the following patient safety incidents: injuries as a result of inappropriate use of physical restraint; respiratory tract infections as a result of swallowing difficulties; and preventable deterioration following illness being mis- or undiagnosed [18].

Following the publication of a report alleging the ‘avoidable’ deaths of people with intellectual disabilities in acute hospitals [19], several inquiries were set up to investigate the healthcare provision for this group [20, 21]. A significant addition to the knowledge base about the contributory factors to the safety risks faced by people with intellectual disabilities within the healthcare system was made by the Confidential Inquiry into Premature Deaths of People with Intellectual Disabilities (CIPOLD) [22], which investigated the events leading up to all known deaths of people with intellectual disabilities in a geographically defined part of England (n = 247) as well as deaths of comparators without intellectual disabilities (n = 58). They found that people with intellectual disabilities experienced significantly more problems with diagnosis and treatment of their illness, as well as with all aspects of care provision, planning, coordination and documentation. Just under half of all deaths of people with intellectual disabilities were avoidable, and more of the people with intellectual disabilities than comparators died from causes that were potentially amenable to change by better-quality healthcare (38% vs 9%) [23].

This paper is based on a study commissioned by the National Institute of Health Research, which aimed to investigate the factors that affect the successful implementation of strategies for improving healthcare provision for people with intellectual disabilities in NHS hospitals [24]. The study focused on strategies that were recommended in the Michael Report, an independent inquiry into access to healthcare for people with intellectual disabilities [20]. The aim of the study was not to quantify the real or potential harm suffered by patients with intellectual disabilities as a consequence of patient safety incidents, nor to make comparisons with the general population, but rather to gain insight into the factors contributing to safety issues encountered by people with intellectual disabilities throughout the hospital pathway.

The aim of this paper is threefold: to describe the challenges in preventing and monitoring patient safety issues for people with intellectual disabilities in NHS acute hospitals, to describe the range of the patient safety issues faced by patients with intellectual disabilities in the study (from those that caused potential harm but no known harm, to those that caused actual harm); and to explore the underlying contributory factors to these safety issues.

## Methods

The study was conducted at six NHS acute hospitals in England over a 21-month period (2011–2013). The sites were purposively selected to cover a range of demographic areas and to vary in size. They comprised two teaching hospitals and four district general hospitals; three were in urban areas, two in urban/rural areas, and one in a rural area. The study obtained ethics approval from London East Research Ethics Committee (reference 11-LO-0428). R&D approval was granted at each of the six research sites. Participants gave their informed consent (or where appropriate a consultee provided assent in line with the requirements of the Mental Capacity Act 2005) to take part in the study. Anonymity and confidentiality was clarified with participants.

The study was concerned with adults with intellectual disabilities (aged 16 and over). Recruitment of participants was facilitated by a study collaborator at each site, who helped to identify potential participants (the nursing director, deputy nursing director, or intellectual disability liaison nurse (IDLN)). This was a complex mixed-method study involving the collection of qualitative and quantitative data, including the following: (a) electronic questionnaires were sent to all clinical hospital staff; (b) semi-structured interviews were conducted with hospital staff including all nursing directors, medical directors and other purposively selected senior managers, and managers of up to three purposively selected wards per hospital as well as nurses on those wards; (c) questionnaires were given or sent to carers of patients with intellectual disabilities during a 12 month data collection period; (d) semi-structured interviews were held with carers who had indicated willingness to be interviewed and provided contact details on the questionnaires; (e) hospital-based expert panels were convened to discuss emerging findings, consisting of purposively selected senior managers and clinicians with knowledge and expertise in providing hospital care to vulnerable groups; (f) incident report data that involved a patient with intellectual disabilities over a 12 month period, as well as data on complaints involving a patient with intellectual disabilities, were scrutinised. Aspects of data collection that involved participants with intellectual disabilities are not reported in this paper. The full methodology and findings are reported elsewhere [24].

Interview schedules and questionnaires were derived from a research framework, which included general queries around the prevention of adverse outcomes as well as a number of questions about specific patient safety issues that had been identified in the literature. A scoping review published at the outset of the research [25] suggested that preventable deterioration and, in particular, medication errors and misdiagnosis (due to problems with communication and comprehension) were specific and pertinent issues faced by patients with intellectual disabilities; these safety issues therefore formed specific lines of enquiry (see Table 1). Staff interviewees were asked to expand on their views and experiences of patient safety incidents, including medication errors and preventable deterioration. Carers were asked to comment on the standard of medical care provided to the patient, on the specific needs of the patient, and on the hospital’s ability to meet those needs. All interviewees were invited to contribute examples of what they perceived as good hospital care, as well as examples of practice where the patient was at perceived risk or had suffered actual harm. Data collection tools were piloted and revised accordingly.

**Table 1:** **Questions within the research framework related to patient safety for people with intellectual disabilities (ID)**

	Organisational context	Staff: individuals and teams	People with intellectual disabilities and carers
**Patient safety/Prevention of adverse outcomes**	● What measures are in place to ensure the safe administration of medication to patients with ID, including giving clear information about medicines to the patient?	● Are individual staff and staff teams aware of the measures to ensure safe administration of medication to patients with ID?	● Do patients with ID and their family/carers think they have been given understandable information about medicines, including medicines to take home?
With specific focus on:			
● Medication errors			
● Preventable deterioration			
		● Are individual staff and teams aware of the measures in place to avoid preventable deterioration and misdiagnosis for patients with ID?	
			● Do patients with ID and their family/carers think that preventable deterioration was avoided?
● Misdiagnosis			
	● What measures are in place to avoid preventable deterioration and misdiagnosis for patients with ID?		
			● Do patients with ID and their family/carers feel they received an accurate and timely diagnosis?
		● Are individual staff and teams aware of the systems in place for reporting adverse outcomes?	
	● What systems are in place for monitoring adverse outcomes and complaints involving patients with ID?		
			● Do patients with ID and their family/carers know how to make a complaint?
		● Are adverse outcomes involving patients with ID reported by staff?	
			● What adverse outcomes and complaints involving patients with ID or their family/carers have been recorded within the hospital during the data collection period?

Data from the two questionnaires were analysed using SPSS Statistics 19 and descriptive statistics were calculated. Analysis of the large amount of qualitative data involved line-by-line coding and was facilitated by the data management programme NVivo 9. The analytic framework included codes for patient safety incidents as well as for participants’ views and experiences with regards to patient safety, and as such enabled the extraction of patient safety incidents throughout the data set. These examples were scrutinised with a focus on determining the contributory factors that underpinned them.

Data analysis was undertaken throughout the study period and with involvement from all members of the research team to ensure reliability. Weekly research team meetings were held to discuss coding and emerging themes, and to amend the coding framework as necessary. Attention was paid to divergent cases. Members of the research advisory board joined in these discussions to add their perspective as needed; this included national patient safety experts, the Chief Executive of an NHS trust, and family carers.

## Results

The results are structured so as to present the findings in relation to recognising and monitoring patient safety incidents for people with intellectual disabilities in NHS acute hospitals, then to describe the range of patient safety issues faced by patients with intellectual disabilities that were captured in the study (from those that caused potential harm but no known harm, to those that caused actual harm), and to explore the underlying contributory factors to these safety incidents. Findings are derived from analysis of the mixed-methods data.

### Participants

A total of 1,251 participants took part; including staff, carers and people with intellectual disabilities (see Table 2 for a detailed breakdown). Four of the six sites were able to host an expert panel discussion.

**Table 2:** **Breakdown of study participants**

Data collection method	Number of participants
**Clinical staff questionnaires (via email)**	
Physicians	159
Nurses	541
HCA	83
AHP	159
Other	48
Not specified	28
Total before exclusions	1018
Excluded	-28
Total	**990**
**Staff Interviews**	
Senior managers	27
Ward manager, matron, senior sister, senior nurse	22
Staff nurse	9
Physicians	5
IDLNs	6
Community ID Nurse	2
Other	6
Total	**77**
**Carer questionnaire**	
Family carers	40
Paid carers	54
Total before exclusions	94
Excluded	-6
Total	**88**
**Carer interviews**	
Family carers	19
Paid carers	18
Total	**37**
**Interviews with people with intellectual disabilities**	**33**
**Patient observation**	
Patient interview/observation	8
Staff interview	8
Carer interview	3
Total	**19**
**Panel Discussion**	
Senior managers	14
Physicians	5
Matrons/Ward Managers/Sister/Ward Nurses	11
Clinical Nurse Specialists	6
IDLNs	2
Community ID Nurses	2
Other	2
Total	**42**
**Total**	**1286**
**Participants counted in more than one data set**	
Carer interviewees also included as carer questionnaire participants	-28
Panel discussion participants also included as staff interviewees	-7
**Total number of participants**	**1251**

### Recognising and monitoring patient safety incidents for people with intellectual disabilities in NHS acute hospitals

#### Incident reports

Obtaining data on incident reports concerning patients with intellectual disabilities was complicated by the fact that the presence of intellectual disability was frequently unidentifiable, which led to one trust being unable to supply relevant incident reports. The remaining five study sites passed on information about a total of 320 incidents (range 13 to 126). It could not be ascertained whether all of these met the inclusion criteria of relating to an “adult patient with intellectual disabilities”. Two trusts supplied incident reports related to patients identified after discharge under ICD code F89.1 “Specific Developmental Disorders of Scholastic Skills”, which is likely to have included patients with conditions other than intellectual disability. The exception was one trust which asked staff to tick a box if the incident involved a patient with intellectual disabilities; however, it is not known how often this was completed correctly as no audit was undertaken. Furthermore, some trusts included incident reports regarding children. Excluding those where the description clearly indicated that the patient was a child, 272 incident reports were included in the analysis.

Assessment of the number of each type of incident reported revealed that the vast majority were about tangible, physical patient safety incidents such as falls (n = 106) and pressure sores (n = 30). The other main category was “physical or verbal abuse to staff” (n = 34). Most incident reports simply described the single event that was, or could have been, constituted as “harm” (such as “patient was found on the floor”, “patient lashed out”), and did not detail the contributory factors leading up to this. The reported incidents are summarised in Table 3.

**Table 3:** **Incidents involving patients with intellectual disabilities, reported through the hospitals’ Incident Reporting Systems**

Number of incidents	Type of incident	Example
106	Falls	“Patient calling out and when entered room found patient on floor.”
34	Physical or verbal abuse to staff	“When going to do an assessment of the patient he grabbed a nurse by the hands and then went to punch me. Security phoned when speaking to the patient after he went for me again. Security had to hold the patient down to the bed so he did not hit anyone.”
*29*	*Patient to staff*	
*5*	*Family/friend to staff*	
30	Pressure sores	“Patient was admitted with stage 2/stage 3 pressure ulcers to her sacrum and a grade 2 pressure ulcer to her ( R ) elbow.”
12	Medication-related	“Drug chart checked prior to administration of Baclofen tablets. Route section of drug chart filled in as oral (O) medication needed to be given via gastrostomy.”
12	Patients absconding / discharging against medical advice	“Patient found wandering around [name of railway station] in underpants and dressing gown with no shoes on.”
10	Feeding-related	“I set a feed up. At the end of his feed I realised that I had given him the wrong feed. He is prescribed [name of feed] and I had actually given him [name of different feed].”
8	Accidents and injuries	“Patient very agitated, nursed on the floor as high falls risk, patient continuously repositioned and nursed in side ward with door open in view of nurses bay. Patient managed to crawl onto floor from floor mattress and hit arm and leg, skin tear to both.”
7	Tracheostomy-related	“Patient has a tracheostomy tube in situ and there was no evidence of tracheostomy care that has been done by the nurses from 2:00 am until the time I saw the patient around 10:00 am. Nothing was documented in the tracheostomy care checklist.”
7	Safeguarding alerts	“Staff within the department raised concerns relating to the patients presenting condition and where concerned that there were issues of self-neglect or neglect by the carers.”
*6*	*Relating to family or community staff*	
*1*	*Relating to hospital staff*	
7	Inappropriate clinical area/ward	“Patient with learning difficulties transferred from CDU to [ward X] despite clear admission criteria regarding [ward X] taking such patients.”
7	Delays to treatment	“Patient with learning difficulties had been admitted due to increasing breathlessness from a large pleural effusion. Due to agitation, it was not safe to perform pleural aspiration or chest drainage under conscious sedation. A decision was taken to perform this under general anaesthesia on the day of admission. The patient was kept nil by mouth for four consecutive days whilst awaiting this procedure. Despite daily communications with the anaesthetic department, the patient did not have this procedure until 5 days post admission.”
*3*	*Capacity and consent issues described as reason for delays*	“Patient arrived in dept for Colonoscopy. Patient restless and wandering around despite carer. No consent filled by home or next of kin and referral does not make clear that the patient has dementia. Carer unable to consent. Patient cancelled as unsafe to do procedure. Patient not understanding anything told to her.”
5	Delays to diagnostic tests	“Ten patients in CDU waiting for x rays. One patient with learning difficulties and aspiration pneumonia has been waiting 3 days for a chest x ray!!! This is not really defensible!!!”
5	Epileptic seizures	“Patient had a seizure attack, fell backwards, fall was broken, fell on bottom.”
16	Other *Includes:* poor record keeping; unavailability of equipment; theft/loss of patient property; patient is upset/shouting; delays in clerking/admission; self harm	“No ECG monitor available in the [theatre X] anaesthetic room to record the ECG tracing when a patient condition deteriorated.”
		“The above patient was a one hour ambulance breach. She arrived in the dept at 15.23 and was not transferred on to a trolley until 16.12.”

#### Complaints

Very limited data were received on formal complaints made by people with intellectual disabilities or their carers. There are three possible reasons for this: (1) there were issues of confidentiality and the information was sensitive, so it was not passed on to the researchers; (2) patients or carers raised their concerns with hospital staff or with PALS, rather than make a formal complaint; (3) patients and carers did not make complaints if they were dissatisfied. There was evidence in the interviews with carers of all of these, and in particular of (2) and (3). Carers who described situations where the patient suffered actual or potential harm were asked for their reasons for not making a formal or informal complaint. They expressed a desire to put the experience behind them as well as concerns that complaining might negatively affect the future health care of the person with intellectual disabilities.

#### Staff perspectives

The clinical staff questionnaire included items related to known safety risks for patients with intellectual disabilities (see Table 4, which presents selected items in relation to patient safety). Staff were asked whether these had occurred within their clinical setting in the past three years (which was the time that had elapsed since the publication of the Michael Report in 2008 [20]).

**Table 4:** **Selected items from the clinical staff questionnaire: frequency and percentage response to patient safety items**

Within your clinical setting, have any of the following occurred involving a patient with intellectual disabilities in the past 3 years?***(number of respondents =825)***	Number (%) indicating ‘yes’ response
Communication with the patient was not as good as it should have been	302 (36.6)
It was not possible to complete a full assessment of the patient’s needs	224 (27.2)
Certain tests or treatments were delayed because the patient was unable to give consent	196 (23.8)
Communication with the family or carers was inadequate	156 (18.9)
It was not possible to obtain advice from a [intellectual] disability expert at the time this was needed	131 (15.9)
Staff avoided the patient because of unusual, different or challenging behaviour	103 (12.5)
Certain tests or treatments were not given because the patient was unable to give consent	71 (8.6)
The patient did not get sufficient food or drink	52 (6.3)
The patient deteriorated unnecessarily	24 (2.9)
The patient was given the wrong medication, the wrong dose, or did not receive their medication	17 (2.1)
The patient was misdiagnosed	10 (1.2)

The staff interview data highlighted that staff did not always interpret patient safety risks and incidents in the same way as the research team. For example, the literature makes clear that ‘preventable deterioration’ and avoidable deaths could occur as a result of a chain of events, including omissions and delays to care and treatment. Staff, however, seemed to interpret this term with regards to acute medical deterioration due to inadequate monitoring of vital signs (leading them to conclude that patients with intellectual disabilities were not at increased risk of preventable deterioration), or safety issues related to the patient being in an unfamiliar environment. The following extracts from three different staff interviews demonstrate this:

**Interviewer:** “Overall what are your thoughts on the safety of care provided for people who have intellectual disabilities?”

**Staff nurse:** “I think it’s the same as the safety for the rest of our patients I don’t think that they are in any further risk than the rest of the patients that we see here.”

**Interviewer:** “What about preventable deterioration, have you ever come across a case where somebody’s health did deteriorate in a way that could have been prevented – with [an intellectual disability]– have you ever come across that on the ward?”

**Senior staff nurse:** Not really. I think everyone is monitored in terms of observations four-hourly on this ward, apart from overnight, and then we do intentional rounds so everybody is checked up on hourly to two-hourly anyway.

**Interviewer:** Do you think there are any specific patient safety issues that people with intellectual disabilities might be particularly vulnerable to?

**Staff nurse:** Um… I suppose if they weren’t supervised it could be a bit of a minefield really. A place like this where there are so many trolleys and equipment and, you know, doors leading to places they shouldn’t go and things like that. So, if they were mobile, yes, I think it could be a bit of a nightmare really.

### Patient safety incidents and contributory factors

Despite the apparent challenges to recognising and monitoring patient safety incidents detailed above, a wide range of patient safety incidents (events that led, or could have led, to the patient suffering avoidable harm) were highlighted by participants in interviews, observations and questionnaires and were evident in incident reports. Synthesis of all data demonstrated that the following safety issues were of particular significance to patients with intellectual disabilities in acute hospitals (not in order of importance or frequency): inadequate provision of basic nursing care; misdiagnosis; delayed investigations and treatment; and non-treatment decisions and Do Not Attempt Cardiopulmonary Resuscitation (DNACPR) orders. Examples of the data are given in Table 5.

**Table 5:** **Patient safety issues and underlying contributory factors highlighted in the study**

Type of issue	Contributory factors	Examples
**Inadequate provision of basic nursing care**	● Lack of staff knowledge or experience to recognise the additional needs of patients with intellectual disabilities	**Staff nurse (free text on clinical staff questionnaire):** ‘I have also seen people avoid feeding these people due to being unfamiliar with them. I have also seen people leave drink and food out of reach for the patient and not offering this to them regularly.’
*for example*	● Staff avoidance of patients with intellectual disabilities due to the perceived additional workload or due to fear of these patients	**Person with intellectual disabilities (interview):** ‘I sneaked off and got a drink. See we were forgotten… three hours later they still ain’t coming with my coffee… it happens quite a lot sometimes. If I was a normal person I’d get treated a bit better, like a proper person. “I can’t deal with this person”.’
● lack of monitoring of patients’ general wellbeing and comfort,		
	● Over-reliance on carers to provide basic nursing care, or incorrect assumptions that carers will do so	**Carer (interview):** ‘The carers were left even to the point of where to get the sheets to change the bed… [the nurses] didn’t come near him, very scared of him.’
● lack of pressure area care.		**Ward manager (interview):** ‘Some nurses shy away from difficult patients. So if there’s a family member there or a carer then they’ll probably quite happily devolve some responsibility to them.’
**Misdiagnosis or delayed diagnosis**	● Diagnostic overshadowing	Example provided by **Director of Operations**: A woman with intellectual disabilities attended Accident and Emergency with a carer. During the triage process, the patient was fiddling with the equipment used to take her observations. The observations were not within the ‘normal’ range and the nurse assumed that this may be because of the patient’s interference. The patient and her carer were requested to sit in the waiting room. The patient deteriorated rapidly during her wait and ultimately died.
		A man with intellectual disabilities attended A&E on his own as he had noticed blood in his underwear. He had difficulty articulating his symptoms and was sent home from A&E as staff incorrectly believed the man was drunk. Later on, a carer noticed the blood and the man returned to A&E. He had a rectal prolapse which required emergency surgery. (Example provided by **Community Intellectual Disability Nurse**)
		**Emergency care practitioner (free text on clinical staff questionnaire): ‘**I once found it difficult to assess a young patient with intellectual difficulties who appeared agitated after a head injury. I had to rely on the information given to me by the mother which was not accurate. The patient was discharged and returned a few hours later with an inter-cranial bleed. This could have been prevented if I had been able to assess the patient better and more thoroughly.’
	● Difficulties in communicating with the patient about symptoms and medical history	
**Delayed investigations and treatment**	● Failure to provide reasonable adjustments to enable the patient to equitably access the service	**Example provided by intellectual disability liaison nurse**: A man with severe autism needed to have an anaesthetic before a procedure in a day clinic. After waiting for two hours, he became so agitated that the planned procedure could not proceed.
	● Communication breakdown with the multidisciplinary team, or between staff and carers, leading to a lack of co-ordination of care	**Radiographer (free text on clinical staff questionnaire):** ‘Frequently, when making appointments, we are not informed that patients have learning disabilities and doctors will request scans which when the patient arrives to have, it is immediately clearly completely inadequate for such a patient to be able to cope with the scan requested and therefore has to be abandoned.’
	● Communication difficulties between staff and patients with intellectual disabilities	**Family carer (interview):** ‘We literally ran round (to try and obtain a timely ‘best interest’ decision to enable her profoundly disabled son to have an urgent procedure to unblock his percutaneous endoscopic gastrostomy (PEG) feeding tube ) What they should understand is that the PEG is his lifeline, the food, water, if that’s not working, he can’t swallow (…) and that’s where we run into trouble. People don’t always get it, they don’t understand that there’s urgency.’
	● Failures in recognising and treating pain	**Person with Intellectual Disabilities:** A couple of times on [the ward] I tried to get their attention, I was in pain and needed medication. I had to get my mum to speak to them and she had to complain, saying I need medication for my pain.
	● Delays due to time taken to establish patient capacity	**Consultant physician (interview):** ‘[The patient] had cancer and needed surgery. I didn’t realise that he didn’t have the capacity to say ‘no’ to the operation. He didn’t want the operation, and I just thought that was that. But [Intellectual Disability Liaison Nurse] came along and asked him, ‘What do you think will happen if you don’t have the operation?’ and he really didn’t know. He didn’t have the capacity. So it became a best interest decision, and we decided to do the operation.’
	● Patients less likely to challenge errors or delays	**Paid carer (interview):** ‘Because the gentleman screams when he is touched, the nurses at the hospital would not touch him. They said ‘no, we can’t, he doesn’t want us touching him’. (…) The doctors and nurses on the ward said ‘the best thing is to let nature take its course and let him die’. This was despite no medical or nursing assessment.’
		**Family Carer (interview):** ‘[The doctor in A&E] took me to one side and he said, “What sort of quality of life is she going to have if we pull her through this?” And I said, “She’ll have a fantastic quality of life, she’s got close family, she’s got excellent carers, she’s got lots of things to look forward to in her life.” And he said, “Well, it’ll be up to the ICU team whether or not they’ll treat her, you do realise that she isn’t going to survive if we don’t treat her?”‘
	● Patient may fail to comply with investigations or treatment	**Paid carer (interview):** ‘If my staff had not supported [patient], he would either be in a coma or dead because they just wouldn’t have given him any medical intervention.’
	● Staff misunderstanding of Mental Capacity Act, or lacking confidence in using it	**Ward manager (interview):** ‘One thing, personally, which upsets me the most – I know they have intellectual disabilities and it’s not very severe sometimes – but they just put all of them when they come in, “Not For Resus”.’
**Non-treatment decisions and DNACPR orders**	● Erroneous staff assumptions about the patient’s quality of life	
	● Staff fear of treating patients who are perceived as ‘challenging’	

### Inadequate provision of basic nursing care

Several carers and hospital staff described a lack of basic nursing care provided for patients with intellectual disabilities, for example a lack of monitoring of patients’ general wellbeing and comfort, insufficient feeding or hydration and lack of pressure area care. Three main contributory factors underpinning these omissions were suggested. Firstly, staff may not have the knowledge or experience to recognise the additional needs of patients with intellectual disabilities. Secondly, staff may avoid patients with intellectual disabilities due to the perceived additional workload or due to fear of these patients. Finally, hospital staff may overly rely upon carers to provide basic nursing care. Inadequate provision of basic nursing care resulted in varying outcomes for the patient ranging from no known harm through to serious harm.

### Misdiagnosis

Examples were given where diagnostic overshadowing (where symptoms are erroneously attributed to the intellectual disability, rather than to a physical illness) had been a contributory factor to misdiagnosis. Difficulties in communicating with the patient about symptoms and medical history were described as being crucial in contributing to misdiagnosis or diagnostic overshadowing. Examples of misdiagnosis of patients with intellectual disabilities had led to varying outcomes, ranging from no known harm through to serious harm and may have been a factor precipitating potentially preventable death (data were unavailable to certify this).

However, it should be noted that a number of hospital staff did not feel that patients with intellectual disabilities were at increased risk of misdiagnosis in comparison to other patients. This finding could, in part, be due to the relative infrequency of misdiagnosis coupled with the relative infrequency of caring for patients with intellectual disabilities. This is perhaps reflected in the results of the clinical staff questionnaire, where only 1.2% of staff (10 out of 825) indicated that a patient with intellectual disabilities had been misdiagnosed within the past three years (see Table 4).

### Delayed investigations and treatment

Several examples illustrated compromised patient safety which resulted in harm to the patient as a result of delayed investigations and delayed treatment. A number of factors underpinned such delays.

In some instances, hospitals had failed to provide the reasonable adjustments (required by law) that are needed to enable the patient to equitably access the service or to receive investigations or treatment. Delays to investigations or treatment had also occurred as a result of communication breakdown within the multidisciplinary team or between staff and carers resulting in a lack of co-ordination of care. Communication difficulties between staff and patients with intellectual disabilities were a particular concern. For example, a number of participants suggested that recognising and appropriately treating pain had been problematic. It was also proposed that patients with intellectual disabilities may be less likely to challenge errors or delays to their care or that they may fail to comply with investigations or treatment, consequently resulting in delays or omissions. Finally, delays to investigations and treatment could be caused by the time taken to establish a patient’s capacity and where necessary to implement the Mental Capacity Act [26].

Within the staff questionnaire, 23.8% of 825 clinical staff indicated that in the past three years tests or treatments had been *delayed* because patients with intellectual disabilities were unable to give consent and 8.6% of 825 clinical staff indicated that certain tests or treatments were *not given* because patients were unable to consent (see Table 4).

Again, delayed investigations and treatment had appeared to result in a range of outcomes for patients with intellectual disabilities, spanning from no known harm through to serious harm.

### Non-treatment decisions and DNACPR orders

Some carers felt that decisions about whether or not to provide active treatment for patients with intellectual disabilities were being inappropriately influenced by staff assumptions about quality of life or by staff members’ fear of treating patients who they perceived to be challenging. In such examples, treatment was provided at the carers’ utmost insistence, and patients who may otherwise have died were able to return home. Similarly, in some cases nursing staff and carers felt that ‘Do Not Attempt Resuscitation’ (DNAR) orders were inappropriately based on staff assumptions about the quality of life of patients with intellectual disabilities. Non-treatment decisions and DNACPR orders led to varying outcomes ranging from mild to moderate through to serious harm to patients with intellectual disabilities.

## Discussion

A wide range of patient safety issues were recounted to the research team and described in incident reports. The key safety issues derived from the analysis were: inadequate provision of basic nursing care, misdiagnosis or delayed diagnosis, delayed investigations and treatment and non-treatment decisions and DNACPR orders. Many of the incidents described were a result of delays or omissions of care or treatment. Such delays and omissions were often underpinned by failures to implement reasonable adjustments for patients with intellectual disabilities (even though these are required by law), a lack of adherence to and understanding of the Mental Capacity Act 2005 [26, 27], failures to listen to carers, staff perceptions and knowledge of intellectual disabilities, and the characteristics of patients with intellectual disabilities which may make them more vulnerable to safety issues. These contributory factors are often inter-related and span organisational, staff/team and patient/carer levels, demonstrating a need for effective policy and a multi-faceted approach to improving the safety of this vulnerable patient group. This is in line with evidence from CIPOLD [22] and other reports and inquiries [17–19, 21], that it is delays and omissions of care that put people with intellectual disabilities most at risk of avoidable harm in hospitals.

The findings of this study demonstrate that one of the central difficulties in monitoring patient safety issues and preventing patient safety incidents for people with intellectual disabilities is the trend for reporting and monitoring those patient safety incidents that result, or could have resulted, in clearly defined physical harm. For example, a fall may result in physical injury, or a patient’s challenging behaviour could result in staff injury; these are therefore recognised and reported as patient safety incidents. However, these *outcomes* are usually preceded by contributory factors. A fall may be due to the patient being confused about an unfamiliar environment, which may be due to a lack of explanations in a way the patient could understand. Therefore, the contributory factors leading up to the harm caused by the fall may have been the failure to provide the reasonable adjustments of extra time, explanations and reassurance. Likewise, a patient hitting others could be a result of earlier events, such as the patient not being given timely medication or nutrition, or having been asked to wait in a busy waiting room. Failure to conduct a timely investigative procedure because a lack of reasonable adjustments could result in a patient being unable to comply or consent which may eventually lead to harm or death. However, such harm is not immediate and therefore may be difficult to attribute confidently to previous incidents, particularly where acts of omission are implicated.

Although acts of omission (failing to diagnose or provide required care) are thought to be twice as prevalent as acts of commission (providing the wrong care) [28] acts of omission are known to be more difficult to recognise, capture and monitor [29]. The study results show that incident reports were strongly biased towards physical patient safety incidents that caused clearly identifiable physical harm. Important issues such as problems with feeding or hydration or delays to patient care and treatment may be less likely to be readily identified as patient safety issues which staff know they are required to report. This highlights the difficulty that acute hospitals may face in monitoring the safety of patients with intellectual disabilities, particularly when incident reporting (which is known to under-represent acts of omission) is overly relied upon as a monitoring method [30].

A further major difficulty in using incident reports to assess safety risks was the lack of effective systems for identifying patients with intellectual disabilities. Furthermore, discussions held throughout the course of the project revealed that hospital staff often felt that the person’s intellectual disability was not relevant to the incident and therefore failed to indicate the intellectual disability on the incident report. For these reasons, incident reports which are specifically flagged as involving a patient with intellectual disabilities are likely to be a small subset of the incidents that actually take place and are consequently a poor method for monitoring patient safety issues in this group of people.

The study findings suggest that there is currently a lack of understanding among hospital staff about the contributory factors which underlie safety issues faced by people with intellectual disabilities. This may reflect a lack of understanding within the NHS that preventable harm for people with intellectual disabilities may result from delays and omissions of care, and that harm may not be immediate or visible. For example, almost a quarter of staff reported in the questionnaire that a patient with intellectual disabilities had experienced delays in treatment of care due to capacity or consent issues. It is now known that this represents a particular and significant safety issue for people with intellectual disabilities, yet there were no mechanisms within the organisation to monitor (and subsequently address) such delays. Only three out of the 272 incident reports in the study related to this kind of incident.

The evidence that people with intellectual disabilities suffer avoidable harm in healthcare is strong [22]. This study has added to the mounting evidence that a lack of reasonable adjustments can lead to delays and omissions of care, and that these can lead to harm.

The patient safety issues and contributory factors presented here may also be of relevance for other vulnerable patient groups, including those with dementia or mental health problems. The challenges in recognising and monitoring safety issues are also likely to persist in other vulnerable groups.

### Limitations

The numbers of carers who participated in this study was relatively low. This was in part due to the difficulties inherent in identifying patients with intellectual disabilities at the point of care. However, this is one of the largest studies to date focusing on the safety of patients with intellectual disabilities in acute hospitals. The number of participants who were carers was large compared to existing studies, and the extended research team agreed that data saturation had been achieved. The staff participants may include over-representation of people with a specific interest in intellectual disability. However, recruitment of a large number of staff enabled capture of a variety of perspectives from people with varying degrees of prior experience.

Additionally, the patient safety issues and contributory factors presented in this paper are of course a non-exhaustive list; patients with intellectual disabilities may experience any manner of patient safety incident and incidents may be triggered by a vast array of factors. Furthermore, this research did not seek to quantify the incidence of patient safety issues experienced by patients with intellectual disabilities in comparison to the remainder of the patient population. Rather, the objective was to describe patient safety issues faced by patients with intellectual disabilities in NHS acute hospitals and the factors that may contribute to these and to consider the ability of acute trusts to recognise and monitor patient safety incidents. Therefore, based on this study we cannot assert that patients with intellectual disabilities experience delays and omissions in their care *more often* than other patients, we can suggest that these issues are a particular concern and that steps should be taken to mitigate the contributory factors identified.

## Conclusion

Patients with intellectual disabilities are vulnerable to delays and omissions of care and treatment in acute hospitals. Acts of omission are often difficult to recognise and capture, therefore it may be difficult for organisations to monitor the safety of patients with intellectual disabilities. Appropriate and active co-ordination of patient treatment and care is therefore vital.

NHS hospitals face significant challenges in identifying and monitoring the healthcare related patient safety incidents that may lead to avoidable harm of vulnerable patient groups such as patients with intellectual disabilities. Drawing on the findings presented in this paper and in the CIPOLD report [22], there is now a need to address the lack of effective monitoring of patient safety for people with intellectual disability at a national policy level. Such monitoring should capture pivotal incidents that are known to lead to potential harm, either immediately or in the longer term. The key to providing safe healthcare for people with intellectual disabilities is the implementation of reasonable adjustments, which requires that (a) the patient is identified within the healthcare system as having intellectual disabilities, and (b) reasonable adjustments are made to the patient’s healthcare delivery [31]. The authors suggest that identifying and documenting the reasonable adjustments needed for each individual patient would go some way in reducing the safety risk of people with intellectual disabilities. A national requirement to do this for all patients, whether disabled or not, subject to inspection and reporting, could make a significant contribution to improving patient safety. In addition, there is a need for acute hospital trusts to ensure that their staff are adequately trained in the use of the Mental Capacity Act and of DNACPR decisions. General staff training to improve understanding of reasonable adjustments and the needs of patients with intellectual disabilities will also help to improve patient safety.

## Abbreviations

A&E: Accident and emergency
CDU: Clinical decision unit
CIPOLD: Confidential inquiry into premature deaths of people with learning disabilities
DNACPR: Do not attempt cardio pulmonary resuscitation
ICU: Intensive care unit
NHS: National Health Service
NPSA: National Patient Safety Agency
PALS: Patient advice and liaison service
PEG: Percutaneous endoscopic gastrostomy
R&D: Research and development
SPSS: Statistical package for the social sciences
UK: United Kingdom
